# Emerging Therapeutic Potential of Mesenchymal Stem Cell-Derived Extracellular Vesicles in Chronic Respiratory Diseases: An Overview of Recent Progress

**DOI:** 10.3389/fbioe.2022.845042

**Published:** 2022-02-25

**Authors:** Yiming Ma, Xiangming Liu, Yingjiao Long, Yan Chen

**Affiliations:** Department of Pulmonary and Critical Care Medicine, The Second Xiangya Hospital, Central South University, Changsha, China

**Keywords:** mesenchymal stem cell, extracellular vesicle, chronic respiratory disease, therapy, microRNA

## Abstract

Mesenchymal stem cell-derived extracellular vesicles (MSC-EVs) are able to carry genetic and protein goods to mediate the interaction between MSCs and target cells. Recently, more and more researches have focused on the therapeutic role of MSC-EVs in chronic respiratory diseases. In this review, we summarize the cumulative strategies and mechanisms of MSC-EVs in treating chronic respiratory diseases. This review suggests that MSC-EVs may serve as a novel cell-free-based therapy for chronic respiratory diseases, including COPD, asthma, pulmonary fibrosis, and pulmonary arterial hypertension. In current studies of chronic respiratory diseases, umbilical cord and bone marrow are main sources of MSC-EVs, while adipose tissue, lung, and induced pluripotent stem cells are also applied. Isolation methods of MSC-EVs in treating chronic respiratory diseases involve ultracentrifugation, exosome extraction kits and anion-exchange chromatography. Intratracheal delivery and intravenous administration are the most widely used routes of MSC-EVs. MSC-EVs are able to transfer microRNAs and protein to target cells and further magnify the therapeutic effects.

## Introduction

Chronic respiratory diseases are diseases of the airways and other structures of the lung, mainly including chronic obstructive pulmonary disease (COPD), asthma, occupational lung diseases and pulmonary hypertension (World Health Organization, 2021). Chronic respiratory diseases remain leading causes of death and disability. It is estimated that 4 million people per year die from chronic respiratory diseases worldwide (Forum of International Respiratory Societies, 2017; Soriano et al., 2020). Considering the heavy social burden from chronic respiratory diseases, effective prevention and treatment measures are urgently needed.

Mesenchymal stem cells (MSCs) refer to a group of cells from bone marrow, fat and umbilical cord, which have the ability of adherent growth. Currently, MSCs are commonly used in cell therapy due to important potentials of proliferation, differentiation and immune regulation (Weiss and Dahlke, 2019). Extracellular vesicles (EVs) are vesicles with membrane structure released by cells in the state of activation, injury or apoptosis; According to the diameter of vesicles, they can be divided into apoptotic bodies, micro vesicles, and exosomes (Shao et al., 2018). A growing number of studies have shown that EVs mediate intercellular targeted regulation by transferring different substances (including protein, DNA, microRNA, lncRNA, circRNA and mRNA) (Lasda and Parker, 2016; Li et al., 2019; Vagner et al., 2019; Vu et al., 2020; Vilaca et al., 2021). Importantly, compared with other commonly used drug delivery carriers (such as liposomes), EVs have the advantages of high internal targeting ability, low immunogenicity, high modification flexibility and high biological barrier permeability, which opens up a new field for modern drug delivery (Herrmann et al., 2021).

MSCs can produce a large number of EVs. MSC-EVs can mediate the interaction between MSCs and target cells through delivering genetic and protein goods, and this targeting mode has attracted great attention in the treatment of chronic kidney diseases, cardiovascular diseases, neurological diseases and tumor (Eirin and Lerman, 19792021; Fu et al., 2020; Tran et al., 2020; Pucci et al., 2021)) MSC-EVs can play a therapeutic role similar to MSCs, and MSC-EVs are easier to store and make dosage forms; Meanwhile, MSC-EVs avoid some limitations of cell therapy, such as embolization and tumorigenesis (Shi et al., 2021). The potential anti-inflammatory effect of MSC-EVs has been proved in several previous studies related to acute lung injury (Monsel et al., 2015; Ahn et al., 2018; Khatri et al., 2018). Recently, more and more researches have focused on the therapeutic role of MSC-EVs in chronic respiratory diseases. In this review, we summarize the cumulative strategies and mechanisms of MSC-EVs in treating chronic respiratory diseases, which may provide novel therapeutic alternatives for chronic respiratory diseases.

## Sources of Mesenchymal Stem Cell-Derived Extracellular Vesicles in Therapeutic Applications of Chronic Respiratory Diseases

Umbilical cord (UC) and bone marrow (BM) are main sources of MSC-EVs in treating chronic respiratory diseases, while other relatively rare sources include adipose tissue, lung, and induced pluripotent stem cells (iPSCs).

UC is an appropriate source of MSC-EVs, with the advantage of non-invasion in the collecting process of UC (de Faria et al., 2012). BM may be suboptimal, as BM-MSCs have shown decreased differentiation potential and BM samples are rather difficult in acquisition (Sergejeva et al., 2004). Of note, the use of adipose tissues to harvest MSCs has become common due to its unique advantages such as easy access, great texture and rapid expansion *in vitro* (Bertassoli et al., 2013; Jalali and Rabiee, 2016). However, few studies have investigated the therapeutic functions of MSC-EVs from adipose tissues in chronic respiratory diseases. Lung is another source of MSC-EVs. Cells derived from human lung allografts present a multipotent mesenchymal cell population, which is locally resident in the human adult lung and has extended life span *in vivo*; And multipotency of these cells was proved by their capacity to differentiate into adipocytes, chondrocytes, and osteocytes (Lama et al., 2007). iPSCs are also sources of MSC-EVs, and MSC-EVs from iPSCs can be utilized for drug delivery by loading with proteins (Yuan et al., 2017). iPSCs maintain the developmental potential to differentiate into advanced derivatives of all three primary germ layers, which provides a novel direction of regenerative medicine.

## Solation Methods of Mesenchymal Stem Cell-Derived Extracellular Vesicles in Therapeutic Applications of Chronic Respiratory Diseases

Methods used to isolate MSC-EVs in treating chronic respiratory diseases involve ultracentrifugation (UCF), exosome extraction kits and anion-exchange chromatography.

UCF is the current gold standard and the most studied methods for EV isolation (Momen-Heravi, 2017). The centrifugal speed for microvesicles is 10,000–60,000 g, while 100,000–200,000 g for exosomes (Borges et al., 2013). However, UCF is time-consuming and isolation outcomes are also highly dependent on speed, rotor type as well as temperature (Livshits et al., 2015). Also, UCF could lead to aggregated EVs after pelleting (Linares et al., 2015). Exosome extraction kits are emerging tools for exosome isolation. One previous study demonstrated that ultracentrifugation and exosome extraction kits are two complementary approaches allowing the detection of different proteoforms with various abundance and purity levels (Gemoll et al., 2020). Thereby, combined isolation methods may be optional to consider in future studies. Anion-exchange chromatography is a scalable protocol developed by Kim et al., in 2016 (Kim et al., 2016). They found that most of the protein from EVs in the harvested medium was bound to an anion exchange resin but that little bound to a cation exchange resin. Besides above mentioned methods, additional techniques or combinations of techniques have been developed to isolate EVs including field-flow fractionation, variations on size exclusion chromatography, microfiltration, asymmetric flow field-flow fractionation, field-free viscoelastic flow, tangential flow filtration and variations thereon, alternating current electrophoretics, deterministic lateral displacement arrays, fluorescence-activated sorting, novel immunoisolation or other affinity isolation technologies etc. (Théry et al., 2018)

## Therapeutic Strategies of Mesenchymal Stem Cell-Derived Extracellular Vesicles in Treating Chronic Respiratory Diseases

The dose of MSC-EVs varies in different disease models. Most studies measured total protein amount using various colorimetric assays to indirectly suggest the particle number of EVs. *In vivo*, 15–100 μg (protein) is the commonly used EV dose for treating mouse or rat models; while 2–40 μg/ml is the usual treatment dose for studies *in vitro*. Some studies used absolute particle count or MSC number to measure EV treatment dose. The absolute particle count of EVs ranges from 2×10^7^ to 2×10^10^ *in vivo*.

Intratracheal delivery and intravenous administration are main routes of MSC-EVs in the treatment of chronic respiratory diseases. Compared with intravenous administration, intratracheal administration is a more direct and accurate treatment mode (Chen et al., 2021). An intraperitoneal route could be selected based on its advantages for multiple or daily treatments of EVs (Maremanda et al., 2019). Details of therapeutic strategies of MSC-EVs in treating chronic respiratory diseases are demonstrated in Table 1.

**TABLE 1:** Therapeutic applications of MSC-derived EVs in chronic lung diseases.

References	Year	Disease	EV source	Isolation method	Therapeutic strategy	Mechanisms
Ridzuan et al. (2021)	2021	COPD	Human UC-MSCs	UCF	*In vivo*: EVs from 2.5 × 10^6^ MSCs over 72 h per rat; once; intratracheal delivery	EVs reduce the pulmonary inflammation by the expression of PRKCZ, and NF-κB subunits p65 and p50
Maremanda et al. (2019)	2018	COPD	Mouse MSCs	Norgen Biotek kit	*In vivo*: 15 μg (protein) EVs per mouse; daily for 10 days; intraperitoneal route *In vitro*: not reported	EVs protect cigarette smoke-induced inflammation and mitochondrial dysfunction
Dong et al. (2021)	2021	Asthma	Human UC-MSCs	exoEasy Maxi Kit (Qiagen)	*In vivo*: 100 μg (protein) EVs per mouse; once; intratracheal delivery In vitro: 10, 20, 40 μg/ml EVs added to macrophages for 24 h	EVs inhibit inflammation by reshaping macrophage polarization via inhibition of TRAF1
Fang et al. (2020a)	2020	Asthma	Human iPSC-MSCs	Anion-exchange chromatography	*In vivo*: 1.5×10^10^ EVs per mouse; once; intravenous administration In vitro: 5×10^8^ EVs added to 3×10^5^ macrophages	EVs ameliorate Th2-dominant allergic airway inflammation through immunoregulation on pulmonary macrophages
Fang et al. (2020b)	2020	Asthma	Human iPSC-MSCs	Anion-exchange chromatography	*In vivo*: 2×10^10^ EVs per mouse; once; intravenous administration In vitro: 5, 40 μg/ml EVs added to ILC2 for 48 h	EVs prevent ILC2-dominant allergic airway inflammation by transferring miR-146a-5p
Du et al. (2018)	2018	Asthma	Human BM-MSCs	UCF	*In vitro*: EVs from 1 × 10^5^ MSCs over 48 h added to 1 × 10^6^ PBMCs for 48 h	EVs upregulate IL-10 and TGF-β1 in PBMCs, and promote proliferation and immune-suppression capacity of Tregs
Zhang et al. (2021)	2021	Pulmonary fibrosis	Rat BM-MSCs	UCF	*In vivo*: 100 μg (protein) EVs per rat; once; intravenous administration	EVs reverse epithelial mesenchymal transition through Wnt/β-catenin signaling
Zhou et al. (2021)	2021	Pulmonary fibrosis	Human BM-MSCs	UCF	*In vivo*: 100 μg (protein) EVs per mouse; once; intravenous administration In vitro: 10 μg EV added to 3–5x10^5^ fibroblast for 24 h	EVs block fibroblast activation and suppress SOX4, DKK1 expression by transferring miR-186
Villamizar et al. (2021)	2020	Cystic fibrosis	Human BM-MSCs	UCF	*In vitro*: 5×10^10^ EVs added to 10^4^ HuBECs for 48 h	EVs deliver a packaged zinc finger activator to HuBECs and activate CFTR
Xu et al. (2020)	2020	Pulmonary fibrosis	Human UC-MSCs	Exoquick exosome precipitation solution (System	*In vivo*: unknown dose; every 4 days; intravenous administration In vitro: not reported	EVs decrease collagen I and fibronectin, and improve pulmonary function
Yang et al. (2020)	2020	Pulmonary fibrosis	Human UC-MSCs	Biosciences)	*In vivo*: 100 μg (protein) EVs per mouse; every 4 days; intravenous administration	EVs inhibit epithelial mesenchymal transition activated by the TGF-β1/Smad2/3 signaling pathway
Wan et al. (2020)	2020	Pulmonary fibrosis	Human BM-MSCs	UCF	*In vivo*: 100 μg (protein) EVs per mouse; once; intravenous administration In vitro: 10 μg EV added to 3–5x10^5^ fibroblast for 24 h	EVs suppress the fibroblast proliferation by downregulating FZD6 expression in fibroblasts via carrying miR‐29b‐3p
Gao et al. (2020)	2020	Pulmonary fibrosis	Rat AMSCs	UCF	*In vivo*: 2.5–2.8×10^10^ EVs per rat; once; intratracheal delivery In vitro: 1×10^9^ EVs added to 1×10^6^ ATII cells for 6 h	EVs inhibit TGF-bRI by transferring let-7d-5p
Zulueta et al. (2018)	2018	Cystic fibrosis	Human lung MSCs	UCF	*In vitro*: EVs from 3 × 10^6^ MSCs over 48–72 h added to 2.5 × 10^5^ HBECs for 30 h	EVs upregulate PPARγ, a transcription factor controlling anti-inflammatory and antioxidant mechanisms via NF-kB and HO-1
Klinger et al. (2020)	2020	PAH	MSCs	UCF	*In vivo*: 100 mg/kg EVs per rat; three times; intravenous administration In vitro: 0, 2, 5, and 10 μg/ml EVs added to macrophages for 48 h	EVs decrease lung macrophages, increase the ratio of M2/M1 macrophages, normalize right ventricular pressure and reduce right ventricular hypertrophy and muscularization of peripheral pulmonary vessels
Zhang et al. (2020)	2020	PAH	Human UC-MSCs	UCF	*In vivo*: 25 μg EV per rat; three times; intravenous administration In vitro: not reported	EVs inhibit hypoxia-induced pulmonary arterial endothelial cell apoptosis, pulmonary arterial smooth muscle cells proliferation, and pulmonary arterioles endothelial-to-mesenchymal transition by upregulating the expression of Wnt5a
Liu et al. (2018)	2018	PAH	Rat BM-MSCs	UCF	*In vivo*: 30 μg EV per rat; once; intravenous administration In vitro: not reported	EVs relieve PAH by regulating renin-angiotensin system
Hogan et al. (2019)	2018	PAH	Human BM-MSCs	Not reported	*In vivo*: 2×10^7^ EVs per mouse; once; intravenous administration In vitro: 2×10^7^ EVs added to PASMCs in a 6-well plate biweekly on culture days 1, 4, 8, and 11	EVs relieve PAH by improving mitochondrial function
Chen et al. (2014)	2014	PAH	Rat BM-MSCs	UCF	*In vivo*: 30 μg (protein) EVs per rat; on alternate days for 2 weeks; intravenous administration	EVs relieve PAH (without specific mechanisms)

MSC, mesenchymal stem cell; EVs-extracellular vesicles; COPD-chronic obstructive pulmonary disease; UC-umbilical cord; PRKCZ -protein kinase C zeta; TRAF1-tumor necrosis factor receptor associated factor 1; iPSC-induced pluripotent stem cell; ILC2-group 2 innate lymphoid cells; UCF-ultracentrifugation; PBMCs-peripheral blood mononuclear cells; Tregs-regulatory T cells; SOX4-sky box transcription factor 4; DKK1-Dickkopf Wnt signaling pathway inhibitor 1; HuBEC-human basal bronchial epithelial cell; FZD6-frizzled class receptor 6; AMSC-adipose derived mesenchymal stem cell; ATII-alveolar epithelial type II; HBEC-human bronchial epithelial cell; PPAR-peroxisome proliferator-activated receptor; PAH-pulmonary arterial hypertension.

## Mechanisms of Mesenchymal Stem Cell-Derived Extracellular Vesicles on Therapeutic Applications of Chronic Respiratory Diseases

### Chronic Obstructive Pulmonary Disease

Previous studies have proved the therapeutic role of MSC-EVs in COPD. Maremanda et al.(Maremanda et al., 2019) reported that exosomes from mouse MSCs could relieve cigarette smoke-induced inflammation and mitochondrial dysfunction in mice and human lung epithelial cells. A recent study demonstrated that EVs from human UC-MSCs were able to reduce both peribronchial and perivascular inflammation, and subsequent microarray analysis revealed that EVs significantly regulate multiple known pathways associated with COPD, including TGF-β receptor signaling pathway, IL-4 signaling pathway, and TNF alpha NF-kB signalling pathway (Ridzuan et al., 2021).

### Asthma

The therapeutic role of MSC-EVs in asthma has been investigated both *in vitro* and *in vivo*. Dong et al. (Dong et al., 2021) found that human UC-MSCs derived EVs inhibited inflammation by reshaping macrophage polarization via blocking tumor necrosis factor receptor associated factor 1 (TRAF1). Fang et al. (Fang et al., 2020a) also investigated effects of MSC-EVs on macrophages in asthma disease model, and their research suggested that EVs from human iPSC-MSCs inhibited the recruitment and polarization of lung macrophages in mice with allergic airway inflammation. Another study by Fang et al. (Fang et al., 2020b) indicated that MSC-EVs prevented group 2 innate lymphoid cells (ILC2)-dominant allergic airway inflammation in mice, and the anti-inflammatory effect might contribute from transferred miR-146a-5p in MSC-EVs. EVs from human BM-MSCs might also have a therapeutic effect on asthma. Du et al. (Du et al., 2018) revealed that human BM-MSCs derived EVs upregulated IL-10 and TGF-β1 in peripheral blood mononuclear cells (PBMCs), and promoted proliferation, immune-suppression capacity of regulatory T cells (Tregs).

### Pulmonary Fibrosis

A growing number of studies have demonstrated that MSC-EVs also play a therapeutic role in pulmonary fibrosis. Xu et al. (Xu et al., 2020) indicated that human UC-MSCs derived EVs could decrease collagen I and fibronectin, and improve pulmonary function. Two Chinese studies proved that EVs from rat BM-MSCs and human UC-MSCs reversed epithelial mesenchymal transition, and the inhibition effect might be related to Wnt/β-catenin signaling and TGF-β1/Smad2/3 pathway (Yang et al., 2020; Zhang et al., 2021).

Three studies indicated MSC-EVs inhibited pulmonary fibrosis by transferring microRNAs. Zhou et al. (Zhou et al., 2021) found that EVs from human BM-MSCs blocked fibroblast activation and suppressed SOX4, DKK1 expression by transferring miR-186. Human BM-MSCs derived EVs could also suppress the fibroblast proliferation by downregulating FZD6 expression via carrying miR‐29b‐3p (Wan et al., 2020). And Gao et al. (Gao et al., 2020) reported that EVs from rat adipose derived mesenchymal stem cells (AMSCs) inhibited TGF-bRI by transferring let-7d-5p in alveolar epithelial cells. MSC-EVs also transferred protein to relieve pulmonary fibrosis. One study by Villamizar et al. (Villamizar et al., 2021) illustrated that EVs from human BM-MSCs were able to deliver a packaged zinc finger activator to human basal bronchial epithelial cells (HuBECs) and activate cystic fibrosis transmembrane conductance regulator (CFTR).

Furthermore, human lung MSC-EVs could upregulate PPARγ, a transcription factor controlling anti-inflammatory and antioxidant mechanisms via NF-κB and HO-1, and prevent cystic fibrosis *in vitro* (Zulueta et al., 2018).

### Pulmonary Arterial Hypertension

Previous studies also investigated the role of MSC-EVs in treating PAH, and the main source of EVs is BM-MSCs. Both rat BM MSC-EVs and human BM MSC-EVs demonstrated therapeutic effects on PAH disease models (Chen et al., 2014; Liu et al., 2018; Hogan et al., 2019), and involved mechanisms included regulating renin-angiotensin system and improving mitochondrial function. Besides, Klinger et al. (Klinger et al., 2020) found that MSC-EVs decreased lung macrophages, increased the ratio of M2/M1 macrophages, normalized right ventricular pressure, and reduced right ventricular hypertrophy and muscularization of peripheral pulmonary vessels. And EVs originated from human UC-MSCs could inhibit hypoxia-induced pulmonary arterial endothelial cell apoptosis, pulmonary arterial smooth muscle cells proliferation, and pulmonary arterioles endothelial-to-mesenchymal transition by upregulating the expression of Wnt5a (Zhang et al., 2020).

**FIGURE 1:**
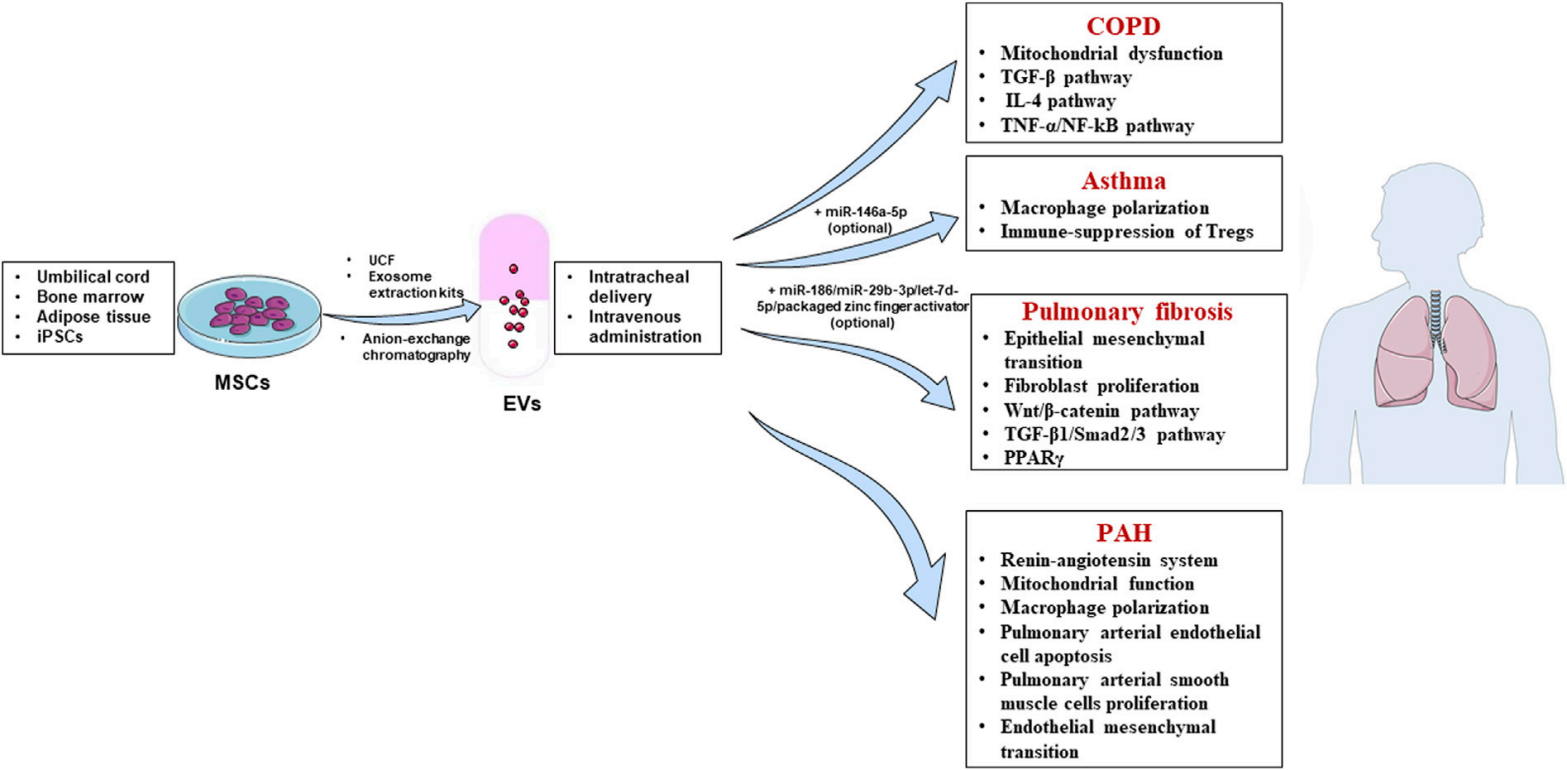
Mechanisms of mesenchymal stem cell-derived extracellular vesicles (MSC-EVs) in treating chronic respiratory diseases. Sources of MSC-EVs: umbilical cord, bone marrow, adipose tissue and induced pluripotent stem cells (iPSCs); Isolation methods: ultracentrifugation (UCF), exosome extraction kits and anion-exchange chromatography; Treatment routes: intratracheal delivery and intravenous administration; Diseases: chronic obstructive pulmonary disease (COPD), asthma, pulmonary fibrosis and pulmonary arterial hypertension (PAH).

Mechanisms of MSC-EVs in chronic respiratory diseases are summarized in Figure 1.

## Conclusions and Future Directions

Collectively, emerging findings reveal that MSC-EVs may serve as a novel cell-free-based therapy for chronic respiratory diseases, including COPD, asthma, pulmonary fibrosis, and pulmonary arterial hypertension. Bone marrow and umbilical cord are main sources of MSC-EVs in treating chronic respiratory diseases, while adipose tissue, lung, and induced pluripotent stem cells are also applied. Isolation methods of MSC-EVs in treating chronic respiratory diseases involve ultracentrifugation, exosome extraction kits and anion-exchange chromatography. As for treatment routes of MSC-EVs, intratracheal delivery and intravenous administration are most widely used. Current studies suggest that MSC-EVs are able to transfer microRNAs and protein to target cells and further magnify the therapeutic effects. However, existing studies have not compared treatment effects among MSC-EVs from different sources (umbilical cord, bone marrow, adipose tissue and induced pluripotent stem cells) or types (apoptotic bodies, microvesicles and exosomes), which highly prompt future studies to address this item. Novel administration routes of MSC-EVs, such as nebulized inhalation, are also worthy of attention so as to improve the safety and effectiveness of MSC-EVs in the treatment of chronic respiratory diseases. Moreover, future studies through nucleic acid sequencing and proteomics are still warranted to reveal internal components of MSC-EVs from various sources and explicit mechanisms of MSC-EVs treatment on different chronic respiratory disease models.

## Abbreviations

MSCmesenchymal stem cell; EVs-extracellular vesicles; COPD-chronic obstructive pulmonary disease; UC-umbilical cord; PRKCZ -protein kinase C zeta; TRAF1-tumor necrosis factor receptor associated factor 1; iPSC-induced pluripotent stem cell; ILC2-group 2 innate lymphoid cells; UCF-ultracentrifugation; PBMCs-peripheral blood mononuclear cells; Tregs-regulatory T cells; SOX4-sky box transcription factor 4; DKK1-Dickkopf Wnt signaling pathway inhibitor 1; HuBEC-human basal bronchial epithelial cell; FZD6-frizzled class receptor 6; AMSC-adipose derived mesenchymal stem cell; ATII-alveolar epithelial type II; HBEC-human bronchial epithelial cell; PPAR-peroxisome proliferator-activated receptor; PAH-pulmonary arterial hypertension.
